# Extracellular Vesicles Regulated by Viruses and Antiviral Strategies

**DOI:** 10.3389/fcell.2021.722020

**Published:** 2021-10-21

**Authors:** Li Yang, Jing Li, Shen Li, Wei Dang, Shuyu Xin, Sijing Long, Wentao Zhang, Pengfei Cao, Jianhong Lu

**Affiliations:** ^1^Department of Hematology, Xiangya Hospital, Central South University, Changsha, China; ^2^Department of Microbiology, School of Basic Medical Science, Central South University, Changsha, China; ^3^National Healthcare Commission (NHC) Key Laboratory of Carcinogenesis, The Key Laboratory of Carcinogenesis and Cancer Invasion of the Chinese Ministry of Education, Cancer Research Institute, Central South University, Changsha, China; ^4^China-Africa Research Center of Infectious Diseases, Central South University, Changsha, China

**Keywords:** extracellular vesicles, virus, isolation, pathogenesis, antiviral therapy

## Abstract

Extracellular vesicles (EVs), consisting of exosomes, micro-vesicles, and other vesicles, mainly originate from the multi-vesicular body (MVB) pathway or plasma membrane. EVs are increasingly recognized as a tool to mediate the intercellular communication and are closely related to human health. Viral infection is associated with various diseases, including respiratory diseases, neurological diseases, and cancers. Accumulating studies have shown that viruses could modulate their infection ability and pathogenicity through regulating the component and function of EVs. Non-coding RNA (ncRNA) molecules are often targets of viruses and also serve as the main functional cargo of virus-related EVs, which have an important role in the epigenetic regulation of target cells. In this review, we summarize the research progress of EVs under the regulation of viruses, highlighting the content alteration and function of virus-regulated EVs, emphasizing their isolation methods in the context of virus infection, and potential antiviral strategies based on their use. This review would promote the understanding of the viral pathogenesis and the development of antiviral research.

## Introduction

Extracellular vesicles (EVs) can be produced by cells and released into the extracellular environment. In the past, the role of EVs had been ignored because they were generally viewed as the waste of host cells. Accumulating studies have demonstrated that EVs could transmit cargo including nucleic acids, proteins, and lipids to mediate the intercellular communication and affect disease physiology (Raposo and Stoorvogel, 2013).

According to their size and origin, EVs are classified mainly into three subpopulations, namely, exosomes, micro-vesicles (MVs), and other vesicles. Exosomes, about 30–150 nm in diameter, are formed through the MVB pathway (Hafiane and Daskalopoulou, 2018). MVs, about 100–1,000 nm in diameter, are produced directly by plasma membrane budding. Other vesicles, such as apoptotic bodies originating from apoptosis cells, are mostly in the micron range (Nagano et al., 2019; Zarà et al., 2019). In general, some molecules, such as CD81, CD9, and annexins, are thought of as the special markers of EVs, including exosomes and MVs (van Niel et al., 2018). However, a recent research has argued about the different components between exosomes and MVs (Jeppesen et al., 2019). Jeppesen et al. (2019) demonstrated that CD63, CD81, and CD9 were the special markers of exosomes. Meanwhile, they emphasized that Annexin A1 existed in MVs, not in exosomes. Therefore, Annexin A1 could be used as a special marker for MVs, filling the gap in the identification of MVs (Jeppesen et al., 2019). Moreover, argonaute proteins, glycolytic enzymes, and cytoskeletal proteins may not be part of exosomes, as described earlier (Ronquist et al., 2013; Melo et al., 2014; McKenzie et al., 2016; Manek et al., 2018; Jeppesen et al., 2019). Thus, further studies about that are needed. Although the size is different between exosomes and MVs, it is relatively difficult for most researchers to separate them completely. Therefore, when exosomes or MVs are isolated from samples, it is more likely to get a mixture of the two (Jeppesen et al., 2019). Meanwhile, the extracellular vesicles 2018 (MISEV2018) guidelines emphasized that the term “EVs” should be used to refer to membrane vesicles isolated by conventional methods (Théry et al., 2018). To be more accurate, exosomes and MVs are referred to as EVs in this review.

Various technologies have been applied to identify the components of EVs and confirmed the presence of proteins, lipids (Schorey et al., 2015), and nucleic acids (Ouyang et al., 2016; Tahamtan et al., 2016). More and more studies have indicated that EVs played a predominant role in regulating the development of diseases by mediating the intercellular transmission of special components. For example, the EVs derived from tumors could limit the immune response (Chen et al., 2018; Gabrusiewicz et al., 2018) or affect the metabolism regulation of the neighborhood cells (Zhang et al., 2017), to remodel the tumor microenvironment.

Viral infection regulates the expression profile of mRNAs, ncRNAs, and proteins in host cells (Kuchipudi, 2015; Tahamtan et al., 2016; Wang, 2019), which affects the gene expression and epigenetic modifications, thus influencing the regulation of the immune response or metabolism (Kuchipudi, 2015; Ouyang et al., 2016; Powdrill et al., 2016). These components can be also packaged into EVs to enhance the intercellular communication of the host system (Raposo and Stoorvogel, 2013); it is not surprising that viruses can take advantage of EVs to promote their infectivity and pathogenicity.

Here, we provide a state of the art regarding the virus-induced modification of EV content and properties. In addition, we also emphasize the isolation methods of these EVs and shed light on their function and the potential antiviral strategies based on EVs.

## The Alteration of Extracellular Vesicles in Components and Number Caused by Virus

### The Altered Host Proteins

Many viruses can dramatically alter the spectrum of host proteins in EVs, and these proteins are mainly related to cellular signaling, immune regulation, metabolism, or autophagy (Meckes et al., 2013; Kalamvoki et al., 2014; Zhao et al., 2014; Shrivastava et al., 2015). In recent years, there have been many other novel finds. The human immunodeficiency virus (HIV) infection of T cells or monocytes and the Ebola virus (EBOV) infection of myeloid cells have also been shown to upregulate certain proteins in EVs; the former could enhance the level of certain cell cycle-related proteins [e.g., cyclin-dependent kinases (CDKs) or high mobility group box 1 (HMGB1) in EVs (Barclay et al., 2019)], and the latter could induce a large number of immune-related molecules [e.g., ribonucleic binding proteins (RBP) and cytokines (Pleet et al., 2018)]. Herpes simplex virus 1 (HSV-1)-infected cells secrete EVs containing stimulator of interferon genes (STING), an innate immune sensor (Deschamps and Kalamvoki, 2018). In addition, hepatitis C virus (HCV) infection could change the expression of transforming growth factor-β (TGF-β) in EVs, thereby promoting the formation of an immunosuppressive environment (Cobb et al., 2018).

### The Altered Host NcRNA

Cell fate is mainly controlled by epigenetics (Li et al., 2019), and ncRNA is one of the classic regulators (Yang et al., 2020). NcRNAs mainly include microRNAs (miRNAs), long non-coding RNAs (lncRNAs), and circular RNAs (Razavi et al., 2021), which play an important role in controlling cell fate (He et al., 2018) and affect the physiology of diseases (Ren et al., 2019; Xie et al., 2019). MiRNAs in EVs are one of the most common targets regulated by viruses, which could assist these viruses to modify the immune response, cause damage to the nervous system, and promote pathological disorders in the body. After the rabies virus (RABV) infection, the expression of miR-423-5p in EVs is upregulated to regulate the interferon (IFN) signaling pathways (Wang J. et al., 2019). MiR-146a (Fu et al., 2017; Santangelo et al., 2018; Wang X. et al., 2019), or miR-148a (Mishra et al., 2020) can also be hijacked by multiple viruses into EVs and contribute to the inflammatory response. The hepatitis B virus (HBV) or HCV infection of hepatocytes could upregulate the level of several miRNAs (e.g., miR-21, miR-29a, miR-19a, miR-192) in EVs to mediate the immunoregulation or hepatic fibrosis (Kouwaki et al., 2016; Devhare et al., 2017; Kim et al., 2019). In addition, HIV can stimulate astrocytes to release EVs containing miR-9, which causes the migration of microglia and neurological disorders (Yang et al., 2018). Japanese encephalitis virus (JEV) infection to microglial cells also increases the expression of let-7a and let-7b in EVs to cause neuronal death (Mukherjee et al., 2019). Moreover, miR-590-5p, an anti-apoptotic molecule, can be more secreted into EVs under the Coxsachie virus B (CVB) infection to play a pro-viral role (Germano et al., 2019).

### The Additional Viral Components

Viruses are so smart that EVs become their tools to transmit their own components. Some viruses, such as HIV, human T-cell leukemia virus (HTLV), Epstein–Barr virus (EBV), or Rift Valley fever virus (RVFV), have been reported to transmit viral proteins into recipient cells through EVs (Meckes et al., 2010; Arenaccio et al., 2014; Jaworski et al., 2014; Ahsan et al., 2016; Arakelyan et al., 2017). Of course, viral nucleic acids are no exception. Interestingly, self-replicating viruses seem to prefer to enclose their genomic RNA into EVs (Ramakrishnaiah et al., 2013; Fu et al., 2017), but retroviruses tend to package their own RNA transcripts (Jaworski et al., 2014; Barclay et al., 2019). Meanwhile, some DNA viruses also take advantage of EVs to load their viral DNA (Mata-Rocha et al., 2019; Sukriti et al., 2019), viral miRNA, or viral mRNA (Meckes et al., 2010; Kalamvoki et al., 2014). Moreover, virus particles can be also wrapped into EVs, which is a common phenomenon for enveloped viruses. Early in 2013, an HCV particle was found in the EVs derived from these virus-infected cells, as determined by transmission electron microscopy (Ramakrishnaiah et al., 2013), and a similar phenomenon was followed by the result of HSV-1 research in the next year (Kalamvoki et al., 2014). Generally, naked viruses spread their virions through dissolving host cells. However, recent studies have also documented that those naked viruses could be transmitted in a non-lysis manner. Santiana and Altan-Bonnet (2019) has revealed that JC polyomavirus (JCPyV) could be released from host cells through the EV pathway. Enterovirus, such as enterovirus 71 (EV71) or echovirus 16 (EV16), also spread their virions in this way (Gu et al., 2020; Netanyah et al., 2020). Therefore, EVs are also one of the effective weapons of naked virus to achieve non-lytic spread and infection.

### The Altered Extracellular Vesicle Number

In addition to the alteration of components in EVs, certain virus infection could also induce the alteration of EV number. Fu et al. (2017) isolated EVs from the supernatants of the mock, EV71, heat-inactivated EV71-infected cells, and determined the EV number; they found that compared with the heat-inactivated EV71 or mock infection group, EV71 infection promoted the EV secretion. Zika virus (ZIKV) usually causes fetal brain abnormalities, but the specific pathogenesis is unclear. A recent study indicated that ZIKV could specifically infect primary human fetal astrocytes and promote the EV release of these cells. After treatment with GW4869, the EV level was decreased and thereby suppressed the ZIKV infection of astrocytes (Huang et al., 2018). In addition, HSV-1 infection could also induce a significant increase of CD63-positive EVs to remodel the extracellular microenvironment (Dogrammatzis et al., 2019). In the study to explore the mechanism of HIV-related central nervous system (CNS) damage, Guha et al. (2019) showed that HIV-positive individuals had more EVs derived from the cerebrospinal fluid, which was positively correlated with neuronal damage biomarkers. Meanwhile, Hinata et al. (2020) also found that EV secretion was upregulated in EBV-infected gastric cancer cell lines.

## The Extracellular Vesicle Isolation From Virus-Infected Samples

The EV size in diameter has been measured and calculated in many studies, which was 30–150 nm for exosomes (Hafiane and Daskalopoulou, 2018) and 100–1,000 nm for MVs (Nagano et al., 2019; Zarà et al., 2019). However, many enveloped and non-enveloped viruses have the same size of that exosome (McNamara and Dittmer, 2020). In addition, EVs and viruses are also similar in density (McNamara and Dittmer, 2020). Therefore, in order to avoid virus contamination, the separation of EVs from virus-infected samples requires more precise separation methods, which is not only a challenge task but also a necessary and critical step for the followed functional studies of these EVs.

Classically, there are five methods based on different theories to isolate EVs from uninfected cells or tissues. There are ultracentrifugation techniques based on the sedimentation rate or density of EVs (e.g., differential ultracentrifugation and density gradient ultracentrifugation), the size-based techniques [e.g., ultrafiltration, size exclusion chromatography (SEC), hydrostatic filtration dialysis], the precipitation-based techniques (e.g., polyethylene glycol (PEG) precipitation and lectin-induced agglutination), the immuno-affinity capture-based techniques, and the microfluidic-based isolation techniques (Li et al., 2017; Doyle and Wang, 2019). Of course, the potential advantages and disadvantages of these techniques have widely been discussed in previous papers, mainly considering the cost of time and expense, the complexity of the procedure, and the yield and purity of EVs (Li et al., 2017; Théry et al., 2018). In recent years, the emergence of commercial kits based on those classical methods has also promoted the development of EV studies. However, Tian et al. (2019) speculated that the EV isolation by commercial kits could result in more non-vesicular contaminants and lower quality compared with differential ultracentrifugation, although a high concentration would be obtained. Therefore, we should take consideration into the quality of EVs when commercial kits are used to isolate EVs. In addition, if EVs are to be functionally verified, highly specific separation methods such as density gradient centrifugation should be applied to separate these EVs (Théry et al., 2018).

**FIGURE 1:**
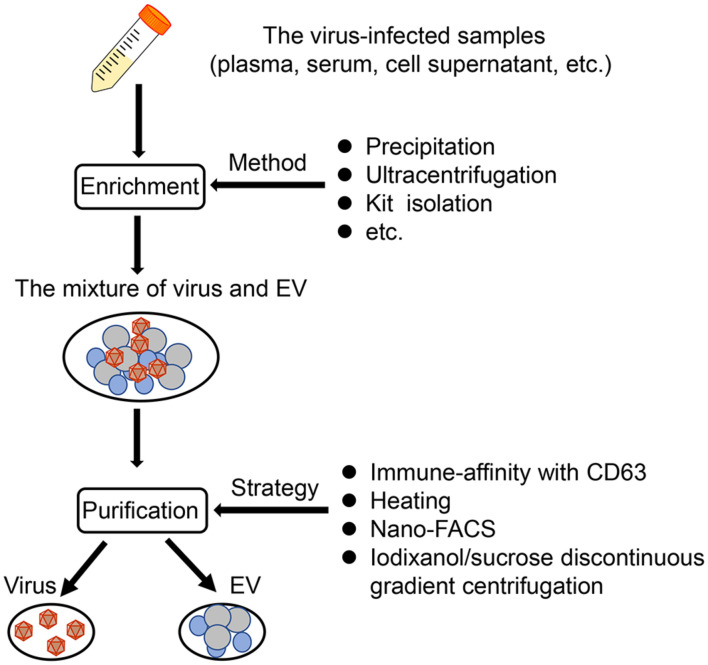
The EV isolation from virus-infected samples. Based on published literature, a synthetic scheme to isolate EVs from virus-infected samples (such as plasma, serum, cell supernatant) is shown here. Generally, there are two steps, namely, enrichment, and purification. First, the mixture of virus and EVs can be obtained through several methods, such as precipitation, ultracentrifugation, and kit isolation. Then, the mixture is purified by applying mainly four strategies, immune-affinity with CD63, heating, nano-FACS, and iodixanol/sucrose discontinuous gradient centrifugation.

Given the truth of similar size and density between virus and EV, it is more difficult and complicated to acquire high-quality EVs from virus-containing samples. Usually, a combination of some methods described above is required for isolating these EVs. Based on the difference of the membrane/capsid composition between virus and EV, affinity purification is a popular manner to further purify EVs following the other isolation methods (e.g., precipitation, commercial EV isolation kit, SEC) (Fu et al., 2017; McNamara et al., 2019; Jung et al., 2020). In addition, the virus-free EVs could be also obtained after applying nanoscale flow cytometry (McNamara and Dittmer, 2020). Adding antigen/substrate specific fluorophores into the mixture of EVs and viruses, and combining with SEC, it is possible to identify and sort EVs specifically (McNamara and Dittmer, 2020). For some researchers with lack of gradient centrifugation and immune-affinity technologies, they can first eliminate the virus from samples based on the different thermal stabilities between some virus and EVs and then purify the EVs through precipitation techniques, just as the isolation of EVs derived from West Nile virus (WNV)-infected cells (Slonchak et al., 2019; Figure 1).

In general, there is currently no uniform method to eliminate the interference of viruses and obtain high-purity EVs. According to the relevant characteristics of the virus, it is wise to combine a variety of separation methods to isolate EVs.

## The Multiple Effects of Virus-Regulated Extracellular Vesicles

### The Effect of Viral Immune Evasion

**FIGURE 2:**
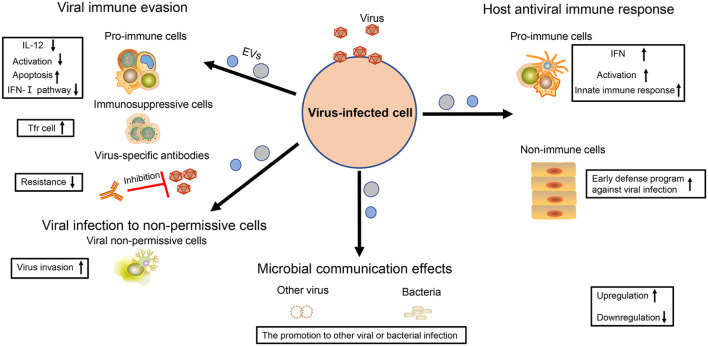
The multiple effects of virus-regulated EVs. The EVs derived from virus-infected cells are endowed with various functions. For virus, these EVs not only contribute to viral immune evasion but also help viruses to infect non-permissive cells. For the host cell, these EVs are also the powerful weapons to achieve antiviral immunity. In addition, the EVs also have an influence on other viral or bacterial infections, thereby contributing to microbial communication.

Some viruses take advantage of EVs to impair the activity of immune cells. It has been shown that some EVs with some RVFV proteins can promote T-cell and monocytic cell apoptosis through PARP cleavage and caspase 3 activation (Ahsan et al., 2016). Furthermore, the EVs carrying EBOV VP40 also cause the apoptosis of T cells (Pleet et al., 2016). A number of studies have also indicated that EV71, HCV, and HBV could force EVs to repress the activation of the type I-IFN pathway in immune cells (Fu et al., 2017; Grünvogel et al., 2018; Shi et al., 2019). In addition, EVs from HBV-infected samples with increased immunosuppressive miRNAs (miR-21, miR-29a) could downregulate IL-12 expression of macrophages and repress the activation of NK cells (Kouwaki et al., 2016), which both weaken the antiviral immune response and accelerate the viral replication. Secondly, some viruses promote the proliferation of immunosuppressive cells and the production of immunosuppressive cytokine. In particular, HCV-infected hepatocytes release the EV packaging with TGF-β to exacerbate the expansion of T follicular regulatory (Tfr) cells, which suppresses the amplification of T follicular helper (Tfh) cells, and the generation of high-affinity antibody-producing B cells (Cobb et al., 2018). In addition, virus particles or viral antigens could be hidden in EVs to avoid to be recognized by the viral specific antibody, which is conducive to the spread of the virus (Ramakrishnaiah et al., 2013; Bello-Morales et al., 2018; Wang T. et al., 2018; Deng et al., 2019; Figure 2).

### The Effect of Viral Infection to Non-permissive Cells

In addition to immune regulation, viruses also utilize EVs to infect the cells without viral receptors, promoting viral spread. Resting CD4^+^ T lymphocytes are resistant to the infection of HIV but become susceptible if they receive the EVs containing HIV Nef (Arenaccio et al., 2014). Meanwhile, it is difficult for HSV-1 to infect Chinese hamster ovary (CHO) cells, but the EVs containing the virus can overcome this problem (Bello-Morales et al., 2018). There is still a gap concerning how JCPyV enters into human brain cells. Recently, two studies have shown that this virus could take advantage of EVs containing JCPyV particles to transmit their infection to cells lacking the virus receptors, thus enlarging virus spread for the development of neurological diseases (Morris-Love et al., 2019; O’Hara et al., 2020; Figure 2).

### The Effects of Host Antiviral Immune Response

As early as in 2012, Dreux et al. (2012) viewed EVs containing HCV-RNA as double-dealers. On the one hand, it is a viral strategy to evade host pathogen sensing. On the other hand, host cells can utilize EVs to activate plasmacytoid dendritic cells (pDC) and induce innate immune response (Dreux et al., 2012). A follow-up study also supported this phenomenon, suggesting that EVs extracted from the supernatant of HBV-infected liver cells were important for NK cell activation, promoting the expression of NKG2D ligands in macrophages and thereby indirectly upregulating the production of IFN-γ in NK cells (Kouwaki et al., 2016). There are innate immune components (e.g., STING) in some EVs regulated by virus, which stimulate the expression of M1-type markers on macrophages to induce the innate immune response and repress viral infection (Deschamps and Kalamvoki, 2018). Moreover, the cells infected with RABV could release miR-423-5p into EVs to upregulate the expression of IFN-β and inhibit RABV replication in turn (Wang J. et al., 2019). Immune cells are very powerful against viral infection, and they can also play an important role with the help of neighborhood cells. For example, when macrophages are infected by dengue virus-2 (DENV-2), they can release EVs containing viral NS3 protein and several special miRNAs to regulate virus-free cells (e.g., endothelium) and to induce their early defense program against viral infection, which could be an alternative strategy for macrophage cells to achieve their immune function (Velandia-Romero et al., 2020; Figure 2).

### The Microbial Communication Effects

In addition to having an influence on themselves, some viruses also further exploit EV function to promote microbial communication. Chen et al. (2020) have shown that HIV-related EVs could enhance the Kaposi’s sarcoma-associated herpesvirus (KSHV) infectivity to human oral epithelial cells. Meanwhile, Hendricks et al. (2021) also reported that the EVs derived from epithelial cells with respiratory syncytial virus (RSV) infection served as the nutrient source of second bacteria (e.g., *Pseudomonas aeruginosa*) and supported their growth (Figure 2). Therefore, these above results indicate that the virus also regards EVs as an effective tool for microbial communication.

## The Pathogenesis of Virus-Regulated Extracellular Vesicles

**FIGURE 3:**
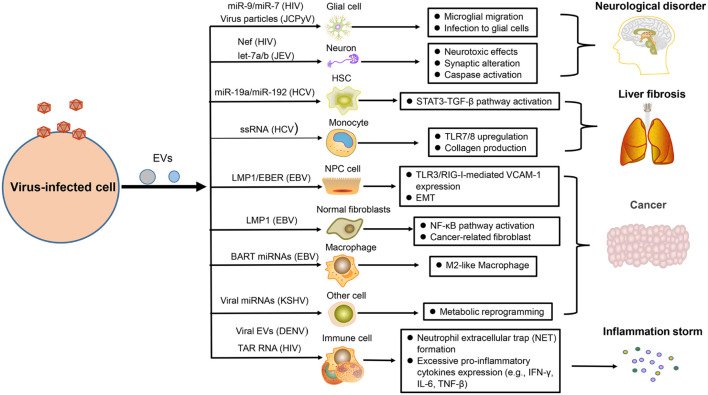
The pathogenesis of virus-regulated EVs. EVs carrying components derived from hosts or viruses under viral regulation can alter the physiological state of the target cells and promote the development of some virus-associated diseases, such as neurological disorder, liver fibrosis, cancer, and inflammation.

With the expansion of human activity, more and more viruses become factors leading to human disease, as far away as smallpox caused by poxvirus (Thèves et al., 2014) and as near as coronavirus disease 2019 (COVID-19) caused by SARS-COV-2 (Lai et al., 2020), which usually brings great negative influence on human health and economy. Therefore, it is essential to understand the viral pathogenesis so as to develop antiviral strategies. The role of EVs in the development of virus-related diseases has been widely discussed in recent years, including their influence on neurological disorders, liver fibrosis, cancers, and inflammation (Figure 3).

### Virus-Associated Neurological Disorders

Many studies have reported that neurological disorders could be attribute to the infection of HIV, EV71, and JCPyV. In combination antiretroviral therapy (cART) for HIV infection, HIV-associated neurological disorders remain a common, and intractable problem. Recently, studies demonstrated that EVs containing HIV-1 Nef could be taken in neurons and induce neurotoxic effects through decreasing glutathione levels or modulating lipid metabolism and lipid raft (Sami Saribas et al., 2017; Ditiatkovski et al., 2020). There are miR-9 or miR-7 in EVs after HIV infection, which separately cause upregulated migration of microglial cells (Yang et al., 2018) or downregulation of neuronal neuroligin 2 (NLGN2) and synaptic alterations (Hu et al., 2019). Microglia are crucial for immune defenses in CNS, but they can also induce neuropathological injury. When microglia are infected by JEV, they can secrete EVs containing let-7a/b into neurons to induce the activation of caspase and cause neuronal death (Mukherjee et al., 2019). Moreover, JCPyV infection is usually fatal for immunocompromised individuals because this virus can explore a variety of pathways to invade CNS and cause progressive multifocal leukoencephalopathy. EV is one of multiple means that mediate the transmission of JCPyV particles among glial cells (Morris-Love et al., 2019; Figure 3).

### Virus-Associated Liver Fibrosis

Liver fibrosis is mainly caused by viral hepatitis, which is an alarm bell of liver injury. To prevent the occurrence of cirrhosis, we need to have a deep understanding of its pathogenesis (Unfried and Fortes, 2020). The EVs produced by HCV-infected hepatocytes not only affect the immune response of hepatic stellate cells (HSC) but also affect their physiological state. Recently, two studies have reported that HSC fibrosis was caused by EVs derived from those cells infected with HCV. Some EVs carry miR-19a or miR-192 to affect HSC fibrosis through activating the STAT3-TGF-β pathway (Devhare et al., 2017; Kim et al., 2019). Some other EVs are packaged with HCV single-stranded RNA (ssRNA) to upregulate the expression of toll-like-receptor 7/8 (TLR7/8) in monocytes, which reinforce collagen production, and induce fibrocystic formation ultimately (Saha et al., 2017; Figure 3).

### Virus-Associated Malignancies

The role of virus in promoting tumor development has earned great attention in the scientific field. EBV is closely related to the occurrence of multiple human tumors, including nasopharyngeal carcinoma (NPC), and lymphoma (Zuo et al., 2017; Li and Lu, 2018). Our result has shown that NPC cells infected by EBV could secrete EVs containing the latent membrane protein 1 (LMP1), which has a positive influence on the epithelial–mesenchymal transition (EMT) of EBV-negative NPC cells (Zuo et al., 2019). Meanwhile, a recent similar result also helped elucidate the relevant oncogenic mechanism of this virus. EVs packaged with LMP1 could transform normal fibroblasts to cancer-associated fibroblasts by affecting the NF-κB p65 pathway, promoting autophagy and aerobic glycolysis. Apart from LMP1, EBV-encoded RNAs (EBERs) could be also transported by EVs derived from EBV-positive NPC to promote angiogenesis *via* affecting the TLR3/RIG-I-mediated vascular cell adhesion molecule 1 (VCAM-1) expression of endothelial cells (Cheng et al., 2019). For supporting the development of lymphoma, EVs with EBV BART miRNAs derived from B-cell lymphoma could promote the tumor pathology by altering the macrophage phenotype (Higuchi et al., 2018). KSHV, another virus closely related to human tumors, could also take advantage of EVs to impact the tumor microenvironment, such as the mediated metabolic reprogramming of adjacent cells (Yogev et al., 2017; Figure 3).

### Virus-Associated Inflammation

As we all know, balanced immunity is important for an effective antiviral response because excessive pro-inflammatory or anti-inflammatory effects will cause pathological damage to the body. However, numerous viruses are able to disrupt the balance of inflammation with the help of EVs and promote the inflammatory state (Duette et al., 2018; Sung et al., 2019). When platelets are infected and activated by DENV, they can drive EVs to activate CLEC5A and TLR2 on neutrophils and macrophages, which can induce neutrophil extracellular trap (NET) formation and proinflammatory cytokine release, causing drastic inflammatory reactions (Sung et al., 2019). For HIV-infected patients, severe inflammation response is a common symptom. A recent report has unraveled that HIV infection could induce the release of EVs containing trans-activation response (TAR) RNA to induce the pro-inflammatory expression of lymphocytes and macrophages, which could contribute to excessive inflammatory response (Sampey et al., 2016; Figure 3).

## Diagnosis and Treatment of Virus-Related Diseases Based on Extracellular Vesicles

### The Potential Biomarker in Diagnosing Viral Infection

**FIGURE 4:**
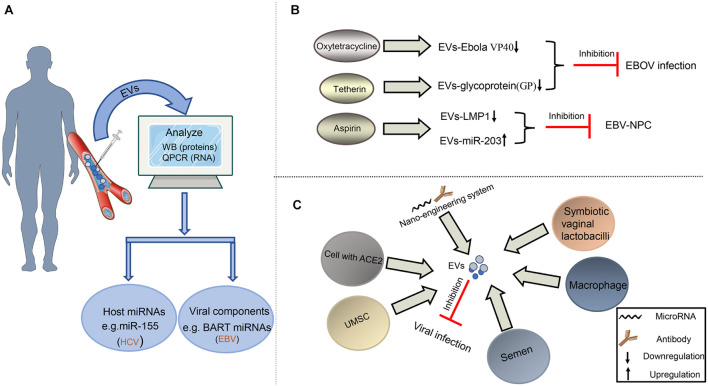
Diagnosis and treatment of virus-related diseases based on EVs. **(A)** EVs as potential biomarkers in the diagnosis of viral infection. EVs from plasma or serum can be extracted and analyzed for the expression level of some special molecules by Western blot or qPCR assays to help the diagnosis of some viral infection. **(B)** EVs as drug target to fight against viruses. Some drugs, such as oxytetracycline, tetherin, and aspirin, may regulate the composition of EVs to inhibit EBOV infection or the development of EBV-NPC. **(C)** EVs as tool to restrict viral infection. The EVs derived from some cells or bacteria carry antiviral factors or viral receptors to limit viral infection. In addition, the artificial EVs produced by the nano-engineering system contain viral antibodies or microRNAs, which can disturb viral replication, survival, and proliferation.

Taking into account the changes in the composition of EVs after virus infection, it provides hope to diagnose viral infection and follow various disease states. Recently, numerous studies have shown that the abnormally expressed host miRNA in EVs could be a potential biomarker of viral infection (Figure 4A). For the rheumatoid arthritis patients, upregulated miR-155 in serum EVs is a potential biomarker of HCV infection (Liao et al., 2018). Similarly, our previous result also concluded that downregulated miR-203 in EVs could be the feature of EBV-NPC (Zuo et al., 2019). In addition to host miRNAs, viral miRNAs, and proteins in EVs also serve as potential biomarkers (Figure 4A). EBV BART miRNAs in EVs may assist to diagnose B lymphoma (Higuchi et al., 2018); HIV Nef packaged into EVs can also contribute to monitoring the development of HIV-related diseases (McNamara et al., 2018).

### Extracellular Vesicles as Potential Target and Tool for Antiviral Therapy

EVs can be the therapeutic targets to resist viral pathology because of their contribution in viral infection and viral diseases. In recent years, researchers have noticed that antiviral drugs could limit the regulatory effects of viruses on EVs (Figure 4B). For restricting EBOV infection, Food and Drug Administration (FDA)-approved oxytetracycline can lower the production of the EVs containing Ebola VP40 to protect the adaptive immune system (Pleet et al., 2016). Moreover, some tetherin can suppress the secretion of EVs with EBOV glycoprotein (GP) by interacting with the GP-transmembrane domain (Nehls et al., 2019). Our previous study on EBV also revealed that aspirin treatment to EBV-associated NPC could downregulate the EV-LMP1 secretion and increase EV-miR-203 expression to inhibit NPC lung metastasis (Zuo et al., 2019; Figure 4B).

In addition to being used as a possible target for antiviral therapy, EVs have become a potential tool to resist viral infection. Earlier reports have shown that semen EVs from healthy human contained antiviral factors, such as host restriction factors (HRFs) mRNAs, and specifically increased resistance to HIV replication and spread (Madison et al., 2014, 2015); so did the EVs derived from symbiotic vaginal lactobacilli (Ñahui Palomino et al., 2019). Meanwhile, the semen EVs with a special lipid fraction are also able to restrict ZIKV transmission (Wang et al., 2020). In the process of suppressing HCV infection, the EVs derived from macrophages or umbilical cord mesenchymal stem cells (UMSC) are an appropriate choice (Qian et al., 2016; Cai et al., 2018; Khatri et al., 2018). At present, COVID-19 caused by SARS-COV-2 is a global public health crisis. It has been shown that the spike (S) protein on this virus surface could bind to angiotensin-converting enzyme 2 (ACE2) of host cells, which promoted the viral entry into host cells (Walls et al., 2020). Cocozza et al. (2020) and Inal (2020) have recently reported that EVs containing ACE2 could block SARS-CoV-2 Spike-dependent infection and could be a potential treatment method for coronavirus infection that rely on ACE2 binding (Figure 4C).

EVs have the advantages of low immunogenicity, high biosecurity, and strong ability to penetrate solid tissue. Thus, it is a kind of nano-engineering system widely used in drug delivery to inhibit viral infection and diseases development (Cheng et al., 2018; Figure 4C). To control the replication of HSV-1 more efficiently, the designed miRNA-401 targeting HSV-1 ICP4 mRNA is packaged into engineered EVs and delivered to viral susceptible cells, which can establish an antiviral environment for at least 72 h (Wang L. et al., 2018). A specific antibody for some viruses, such as HIV and human papillomavirus (HPV), can be also packaged into engineered EVs to selectively target these infected cells (Ferrantelli et al., 2019; Zou et al., 2019). Owing to the placenta barriers and high security requirements, the treatment for fetal defects caused by ZIKV remains a global public concern. Zou et al. (2021) produced an engineered EV carrying interferon-induced transmembrane protein 3 (IFITM3), which could pass through the placental barrier and suppress ZIKV infection in the fetuses of pregnant mice.

## Conclusion and Perspective

EVs are the group of cell-derived vesicles, including exosomes and MVs, which are regarded as the important mediator of intercellular communication and have potential clinical application value. As an important pathogenic factor for human beings, viruses have been shown to regulate various cellular mechanisms. Virus can regulate the EVs to mediate immune evasion and virus spread, causing the development of various diseases, including cancers and neurological disorders. However, EVs also play a role in immune resistance to viral infection, which is a strategy of host antiviral effect. Moreover, EVs regulated by virus also play an important role in promoting microbial communication and mediating the infection of other virus or bacteria.

Although many different components in EVs have been observed after virus infection, the function of these altered components, especially of the altered host components, remains unclear in most extent. Thus, many efforts will be required to resolve this problem in the future. Given the truth that the diameter of EVs is similar to that of most viruses, the separation technology of EVs is really crucial, which is the premise of analyzing the composition and function of these EVs.

Extensive studies have shown that EVs could serve as potential biomarkers and therapeutic targets of some viral diseases. In terms of the latter, some related drugs can be used to change the number and component of EVs and restrict the feature of virus. However, the drugs targeting the virus-regulated EVs are scant. In order to promote the research of antiviral drugs, we should further clarify how viruses interfere with the biosynthetic mechanism of EVs. In addition, we can also take advantage of EVs with antiviral elements to resist the viral infection. Some EVs from body fluids, cells, and even bacteria could have the ability as engineered EVs to inhibit viral infections. Of course, it may be expected to treat COVID-19 caused by SARS-COV-2 if some strategies based on EVs are taken.

## Author Contributions

LY and JL performed the selection of literature, drafted the manuscript, and prepared the figures. SL, WD, SX, SJL, WZ, and PC collected the related references and participation in discussion. LY and JHL designed this review and revised the manuscript. All authors contributed to this manuscript, read and approved the final manuscript.

## Conflict of Interest

The authors declare that the research was conducted in the absence of any commercial or financial relationships that could be construed as a potential conflict of interest.

## Publisher’s Note

All claims expressed in this article are solely those of the authors and do not necessarily represent those of their affiliated organizations, or those of the publisher, the editors and the reviewers. Any product that may be evaluated in this article, or claim that may be made by its manufacturer, is not guaranteed or endorsed by the publisher.
